# Variant Curation is Crucial to Claim Digenic Inheritance in Juvenile Open Angle Glaucoma

**DOI:** 10.1055/s-0041-1733963

**Published:** 2021-08-19

**Authors:** Kok-Siong Poon, Karen Mei-Ling Tan

**Affiliations:** 1 Department of Laboratory Medicine, National University Hospital Singapore, Singapore

The genetics of juvenile open angle glaucoma (JOAG) is heterogeneous in association with multiple loci. 1 2 3 4 A recent case report described a family in which multiple members were affected by JOAG with autosomal dominant (AD) inheritance. 5 In the study, the causative genetic variants were first elucidated by whole exome sequencing in the proband and her affected father, and were further confirmed by targeted Sanger sequencing in her two siblings with ocular hypertension. Pathogenic variants in the *MYOC* gene encoding myocilin is the most common cause of AD form of JOAG. 6 7 In this family, the *MYOC* p.Pro370Leu variant, which is a known pathogenic variant, 8 9 was found in all affected family members. An additional missense variant, p.Pro432Leu in the *LTBP2* gene, was also observed to co-segregate with *MYOC* p.Pro370Leu. Interestingly, digenic inheritance of the *MYOC* and *LTBP2* genes located at 1q24.3 and 14q24.3, respectively, was proposed by the authors to delineate the genetic cause of JOAG in this family.

Suggestion of digenic inheritance in JOAG is not new. A heterozygous variant in *CYP1B1* , which is known to cause an autosomal recessive (AR) form of JOAG, 10 was reported as a modifier gene when inherited together with a *MYOC* pathogenic variant, predisposing the affected family members to earlier disease onset. 11 Similarly, biallelic variants in the *LTBP2* gene cause an AR form of JOAG. 12 The authors postulated that *MYOC* p.Pro370Leu might be necessary for disease manifestation in one of the healthy family members who only carries heterozygous *LTBP2* p.Pro432Leu. It was also hypothesized that with *LTBP2* p.Pro432Leu alone, the carrier may have late disease onset since manifestation of JOAG can be progressive. When coinherited with *MYOC* p.Pro370Leu, the *LTBP2* p.Pro432Leu was also deduced to act as a modifier allele instead of being disease-causing on its own.

Digenic inheritance occurs in a disease context when variant genotypes at two loci explain the phenotypes of some patients more clearly than the genotypes of one locus alone. 13 While the *MYOC* p.Pro370Leu variant in comparison with other *MYOC* variants is associated with early onset, severe disease, and having a penetrance of more than 75% by the age of 25, the authors argue that the family members with both the *MYOC* and *LTBP2* variants have a penetrance of 100% by the age of 15 suggesting the *LTBP2* p.Pro432leu enhances the pathogenicity of the *MYOC* variant. The evidence of pathogenicity of the *LTBP2* variant was substantiated by the authors based on computational prediction by DEOGEN-2 and FATHMM-MKL although other predictors including SIFT and PolyPhen-2 did not predict it to be deleterious. These postulations prompted us to further curate the *LTBP2* p.Pro432Leu variant to classify its pathogenicity.

Although classified as a disease mutation in Human Gene Mutation Database Professional 2020.4, 14 Abouelhoda et al found *LTBP2* p.Pro432Leu in homozygosity in normal individuals from Saudi Arabian populations enriched for homozygosity due to inbreeding. 15 The variant has a relatively high minor allele frequency (2%) in the Indian ethnicities according to 1,000 genomes project phase III. The latest classification in ClinVar interpreted this variant with conflicting interpretations of pathogenicity, which are aggregated from four submissions including two “benign” and two “uncertain significance”. Finally, we applied the American College of Medical Genetics and Genomics/Association for Molecular Pathology criteria 16 using VarSome version 9.2.0 17 and found that it was predicted to be “likely benign” since only PM2, BP1, and BP4 criteria are met.

Collectively, the clinical significance of p.Pro432Leu variant remains unclear. In view of the conflicting evidence, the involvement of *LTBP2* in digenic inheritance of JOAG warrants further investigation. Reporting additional variants with conflicting interpretations of pathogenicity may complicate genetic counseling or raise unnecessary anxiety in the carriers of this variant on the risk of developing genetic eye diseases.
